# The Effects of Weight Perception on Adolescents’ Weight-Loss Intentions and Behaviors: Evidence from the Youth Risk Behavior Surveillance Survey

**DOI:** 10.3390/ijerph121114640

**Published:** 2015-11-17

**Authors:** Maoyong Fan, Yanhong Jin

**Affiliations:** 1 Economics Department, Ball State University, Muncie, Indiana, USA; E-Mail: mfan@bsu.edu; 2 Department of Agricultural, Food and Resource Economics, Rutgers University, New Brunswick, 08901, New Jersey, USA

**Keywords:** childhood obesity, overweight perception, weight-loss behavior, diet habit, physical activity

## Abstract

The objective of this study was to examine the correlation between self-perception of being overweight and weight loss intentions, eating and exercise behaviors, as well as extreme weight-loss strategies for U.S. adolescents. This study uses 50,241 observations from the Youth Risk Behavior Surveillance Survey (YRBSS) 2001–2009, which were nationally representative sample of 9th- through 12th-grade students in both public and private schools in the US. This study finds that, irrespective of the weight status base on self-reported weight and height, adolescents who perceive themselves as overweight have a stronger intention to lose weight, but do not develop better eating and exercise habits, compared with their counterparts of same gender and reported weight status. Normal-weight adolescents, if they perceive themselves as overweight, are more likely to engage in health-compromising weight-loss methods. This study shows that it is critical to transform weight-loss intentions into actual behaviors among overweight/obese adolescents and improve the efficacy of behavioral interventions against childhood obesity. It also highlights the need of establishing a correct perception of body weight among normal weight adolescents to curb extreme weight-loss methods.

## 1. Introduction

Childhood obesity has significantly negative social and health consequences during childhood [1] and leads to adulthood obesity [2]. The fast growing obesity rate among U.S. adolescents, which has quadrupled from 5% in 1980 to 18% in 2009 [3], is largely attributable to unhealthy lifestyle, including poor diet and physical inactivity [4]. Obesity prevention aiming to improve lifestyle, particularly, eating and exercise behaviors, however, has had limited success [5,6].

Distorted self-perception of weight status is one of the important factors leading to low motivation or self-esteem and consequently the ill-fated intervention programs [7,8]. For example, events that promote perception of competence will increase a person’s intrinsic motivation [9]. Overweight individuals need to recognize that their weight status is hazardous to health before they are motivated to make any changes [10,11,12]. Normal-weight adolescents with weight misperception may engage in potentially harmful behaviors such as purging, using laxatives, taking diet pills, and fasting [13,14,15] and expose themselves to a higher risk for eating disorders and depression [10,16]. The literature shows that U.S. adolescents do not necessarily correctly perceive their weight status [17,18,19]. Few studies distinguish between weight-loss intentions and behaviors although they can be significantly different as predicted by planned behavior theory [20]. A majority of studies fail to control for necessary confounding variables that are associated with both weight perception and weight-control behaviors (e.g., depression).

The objective of this study was to examine the correlation between self-perception of being overweight, and weight-loss intentions, eating and exercise behaviors, as well as extreme weight-loss strategies for U.S. adolescents. Using the Youth Risk Behavior Surveillance Survey (YRBSS) 2001–2009 and non-parametric matching technique, we find that, irrespective of gender and self-reported weight status, adolescents who perceive themselves as overweight have a stronger intention to lose weight, but fail to improve their eating habits such as consuming more fruits and vegetables and fewer soft drinks and engage in more physical activity and less sedentary activity. The gap between weight-loss intentions and behaviors suggests that misleading conclusions might be drawn if researchers do not distinguish between them. We also find that the impacts of the perception of being overweight differ between normal-weight and overweight adolescents. Compared with overweight adolescents, normal-weight adolescents have a much greater intention to lose weight if they perceive themselves as overweight and a much higher probability of adopting extreme weight-loss methods. The results indicate that having a correct self-perception of weight status is critical to the success of education and behavior intervention programs for both overweight and normal-weight adolescents as well as to the long lasting program effect on actual weight improvements and healthy lifestyles.

## 2. Methods

### 2.1. Data

YRBSS includes a national school-based survey conducted by the CDC and state, territorial, tribal, and local surveys conducted by state, territorial, and local education and health agencies and tribal governments. The YRBSS is a nationally representative sample of 9th- through 12th-grade students in both public and private schools in the United States. The YRBSS surveys started in 1991 and have been conducted every two years by CDC. In each survey year more than ten thousand adolescents were surveyed. This study excludes the earlier waves of the YRBSS data of 1991, 1993, 1995, 1997 and 1999 either because BMI information or important matching variables (e.g., average GPA) were not available. This study uses the YRBSS data of 2001, 2003, 2005, 2007, and 2009. A total of 50,241 YRBSS observations are included for this study. We incorporate sampling weights in all analyses.

### 2.2. Control Variables

The YRBSS respondents were asked to report their height and weight without shoes on, which are used to calculate their BMI and classify their self-reported weight status. An adolescent is classified as overweight (obese) if his/her BMI is at or above the 85th (95th) percentile for his/her age and gender. Table 1 provides summary statistics of the observations by gender and their reported weight status (normal weight *versus* overweight). Female adolescents account for a slightly higher proportion (52%). They are equally distributed in grades 10–12 and the average age is approximately 16 years old. The BMI category is coded based on the BMI percentile. It equals one for the 5th percentile, two for the 5th–15th percentiles, and so on until eleven for above the 95th percentile. The summary statistics on the BMI category suggests that overweight adolescents have a significantly higher BMI than their counterparts. Non-Hispanic whites account for more than half in the normal weight adolescents and Hispanics and African blacks account a significant proportion in the overweight subsample (56.4% for females and 47.0% for males) for both males and females. The average GPA, which is a proxy for cognitive ability and the awareness of nutrition and physical activity, is found to be slightly higher for overweight individuals than normal weight ones. In terms of risk behaviors, we construct two depression-related variables indicating whether the respondent felt sad (*depression*) and made a suicide plan or attempted to commit suicide (*suicide*). We also create variables to describe the history of smoking, drinking, drug usage, driving under influence, and sexual activities: whether the respondent ever smoked (*smoke*) because smoking may be a causal factor of obesity according to some studies [21]; had alcohol drink at least once for more than five days in the past 30 days (*drink*) because binger drinking is found to be associated with obesity [22]; ever used marijuana (*marijuana*); drove under influence or rode with a driver under influence (*DUI*) in the past 30 days; and ever had sexual activities (*sex*) because they are likely to be related to self-perception of body image and therefore eating and exercise behaviors. Compared with the normal weight individuals, overweight adolescents face a greater risk of depression and attempting/planning for suicide and they are more likely to have smoking history and sexual activities for both females and males.

**Table 1:** Summary Statistics of Control Variables for the YRBSS Samples.

	Female		Male	
	Self-Reported Normal-Weight	Self-Reported Overweight	Self-Reported Normal-Weight	Self-Reported Overweight
BMI category	6.044	10.392	6.184	10.484
	(2.294)	(0.488)	(2.309)	(0.500)
Age (years)	16.136	16.082	16.273	16.200
	(1.198)	(1.238)	(1.202)	(1.216)
Non-Hispanic White	0.524	0.374	0.539	0.451
	(0.499)	(0.484)	(0.498)	(0.498)
Hispanics	0.240	0.297	0.229	0.298
	(0.427)	(0.457)	(0.420)	(0.458)
African American	0.153	0.267	0.150	0.172
	(0.360)	(0.442)	(0.357)	(0.378)
GPA	2.127	2.465	2.178	2.424
	(0.908)	(1.001)	(0.916)	(0.953)
10th grade	0.249	0.244	0.244	0.25
	(0.432)	(0.430)	(0.430)	(0.433)
11th grade	0.262	0.247	0.258	0.255
	(0.440)	(0.431)	(0.438)	(0.436)
12th grade	0.261	0.248	0.273	0.257
	(0.439)	(0.432)	(0.446)	(0.437)
Depression	0.346	0.402	0.205	0.217
	(0.476)	(0.490)	(0.404)	(0.412)
Suicide	0.173	0.224	0.105	0.113
	(0.378)	(0.417)	(0.307)	(0.316)
Sex	0.484	0.492	0.519	0.533
	(0.500)	(0.500)	(0.500)	(0.499)
Drink	0.259	0.236	0.309	0.308
	(0.438)	(0.425)	(0.462)	(0.462)
Smoke	0.522	0.603	0.576	0.599
	(0.500)	(0.489)	(0.494)	(0.490)
Marijuana	0.375	0.407	0.464	0.459
	(0.484)	(0.491)	(0.499)	(0.498)
Drive	0.344	0.327	0.339	0.351
	(0.475)	(0.469)	(0.473)	(0.477)
Observations	19,291	6833	16,258	7859

Notes: Numbers above parentheses are the mean and numbers in parentheses are the standard deviation for each variable by gender and self-reported weight status.

The respondents were also asked to describe their weight using a 5-point Likert-type scale: very underweight, slightly underweight, about the right weight, slightly overweight, and very overweight. We then classify their perceived weight status as overweight if they choose one of the last two options (slightly overweight or very overweight) and normal-weight if they choose one of the first three options.

**Figure 1:**
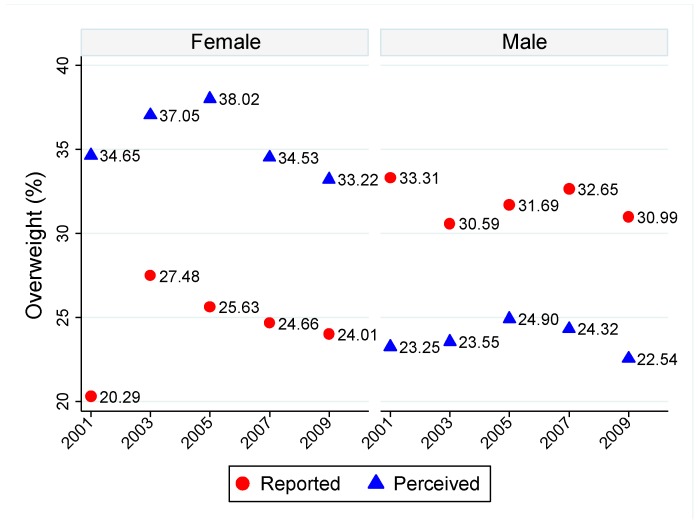
Prevalence of Reported and Perceived Overweight by Gender and Year.

Figure 1 shows statistically significant and persistent discrepancies of obesity prevalence based on perceived and reported overweight status. The self-perceived overweight prevalence is higher (lower) than reported overweight prevalence for females (males). This is consistent with the literature as female adolescents are found to be less satisfied with their body weight and want to be thinner, while male adolescents prefer to increase muscle tone [23].

### 2.3. Outcome Variables: Weight-Loss Intensions and Behaviors

Appendix A provides the details on how outcome variables are created base on YRBSS questions. We distinguish weight-loss intentions and behaviors as our empirical results show that the claimed weight-loss behaviors are dramatically different from what the respondents actually do. We use the word “intentions” for claims made by the respondents about their weight-loss behaviors. The three variables for weight-loss intentions indicate whether the respondent tried to lose weight (*LoseWeight*) and whether the respondent specifically did so through eating less food and fewer calories (*LoseWeight_Diet*) or exercise (*LoseWeight_Exer*) during the past 30 days. As shown in Table 2, for both normal-weight and overweight subsamples, adolescents with overweight perception have a statistically stronger intention to lose weight or keep from gaining weight in general through either diet or exercise than those who perceive themselves being non-overweight (e.g., compare Columns 1 and 2 for the reported overweight females). Second, weight-loss intentions are similar for the reported normal-weight and overweight subsamples if them perceive themselves as overweight (e.g., compare Columns 2 and 5 for females), but are significantly different between two subsamples if they do not perceive themselves as overweight (e.g., compare Columns 1 and 4 for females).

**Table 2:** Summary Statistics of Weight-Loss Intentions and Behaviors by the Reported and Perceived Weight Status (in percentage).

	Females						Males					
Reported overweight (Yes/No)	Yes			No			Yes			No		
Perceived overweight (Yes/No)	No	Yes	*P* ^a^	No	Yes	*P* ^a^	No	Yes	*P* ^a^	No	Yes	*P* ^a^
	(1)	(2)	(3)	(4)	(5)	(6)	(7)	(8)	(9)	(10)	(11)	(12)
	**Panel A: Weight-loss Intentions**											
LoseWeight_Diet	53.12	75.62	0.00	43.39	77.80	0.00	31.06	58.70	0.00	15.65	53.70	0.00
LoseWeight_Exer	68.97	80.98	0.00	60.61	81.77	0.00	63.33	80.30	0.00	40.88	74.42	0.00
	**Panel B: Eating Habits**											
Fruit_2	18.07	17.22	0.50	17.76	17.70	0.93	21.09	16.40	0.00	17.79	17.10	0.60
Vegetable_3	6.12	4.96	0.16	4.49	4.81	0.51	8.09	4.80	0.00	5.06	6.29	0.17
Soda	29.83	28.35	0.58	25.45	23.93	0.27	36.82	35.67	0.50	36.36	39.10	0.26
	**Panel C: Physical and Sedentary Activity**											
Active_5 Days	26.64	20.75	0.00	29.99	26.17	0.00	50.19	35.20	0.00	47.82	33.32	0.00
Moderate_Exer	20.00	21.47	0.24	24.70	24.61	0.91	30.24	25.19	0.00	30.57	25.80	0.00
Vigorous_Exer	51.82	52.65	0.61	58.56	60.00	0.18	75.70	68.80	0.00	74.79	65.56	0.00
PE_Class	55.66	49.64	0.00	52.04	51.46	0.65	64.28	57.05	0.00	59.67	55.02	0.01
Sport_Team	43.34	40.45	0.06	54.15	53.62	0.61	70.04	54.60	0.00	64.53	52.91	0.00
TV_Time	48.83	42.22	0.00	33.39	31.25	0.05	41.49	45.92	0.00	35.90	39.41	0.04
Video_Time	18.77	21.09	0.10	17.07	18.29	0.20	28.18	30.94	0.05	26.30	30.49	0.02
	**Panel D: Unhealthy Weight-loss Methods**											
LoswWeight_Fasting	18.30	23.72	0.00	11.85	28.28	0.00	8.67	11.19	0.00	4.72	15.02	0.00
LoseWeight_Med	11.61	21.10	0.00	8.31	22.03	0.00	8.09	9.79	0.05	3.63	10.73	0.00

Notes: ^a^ *P*-value of t tests for the equal mean of weight-loss intentions and behaviors between perceived overweight and normal-weight adolescents.

The 2010 Dietary Guidelines for Americans promote fruit and vegetable intakes as they are found to be associated with a decreased risk for various chronic diseases and help aid in weight management [24]. However, the CDC estimate that 28.5% of high school students consumed fruit less than one time daily and 33.2% consumed vegetables less than one time daily. The YRBSS respondents were asked to report the number of servings for fruits and vegetables as well as the number of soft drinks excluding diet coke and diet pop during the past seven days. We create two dummy variables indicating whether the respondent meets the recommended consumption levels of fruits and vegetables: ate vegetables at least three times per day (*Vegetables_3*) and ate fruits at least twice per day (*Fruit_2*) during the past seven days. The third eating behavior variable is about consumption of soft drinks (*Soda*) which indicates whether the respondent consumed soft drinks, excluding diet coke and diet pop, at least once a day during the past seven days. As shown in Table 2, self-perception of being overweight is not associated with a higher probability of meeting the recommended consumption levels of fruits and vegetables or lower soft drink consumption.

Physical activity is found to have health benefits [25] and should be considered when addressing weight management [24] Yet, almost a quarter (23%) of 9th- through 12th-grade students did not meet the level of at least 60 min of physical activity daily that is recommended by the 2008 Physical Activity Guidelines for Americans [26]. Based on the relevant YRBSS questions, we create five variables related to personal exercise habits and two variables for sedentary activities (see Appendix A for details).

These variables concern whether the respondent in the past seven days was physically active for at least 60 min on at least five days (*Active_5 Days*), engaged in vigorous exercise that made them sweat and breath hard for at least 20 min on at least three days (*Vigorous_Exer*), or engaged in moderate exercise that did not make them sweat and breath hard for at least 30 min on at least five days (*Moderate_Exer*). We also create two dummy variables for team exercise indicating whether the respondent attended physical education classes on at least one day in an average week (*PE_class*) and played on at least one sport team in the past year (*Sport_Team*). Two dummy variables, indicating whether the respondent had watched television (*TV_Time*) or played video or computer games for more than three hours (*Video_Time*) on an average school day, are also included to measure sedentary activity. As shown in Table 2, compared with adolescents who do not perceive themselves as overweight, those who do are less likely to engage in either personal or team exercise, but more likely to watch TV or play video games.

We also consider two unhealthy weight-loss methods: going without eating for at least 24 hours, *i.e.*, fasting, (*LoseWeight_Fasting*) and taking diet pills, powders, or liquids without a doctor’s advice (*LoseWeight_Med*). These two extreme weight-loss methods are associated with medical complications such as cardiac problems and refeeding complications [27] as well as severe, potentially life-threating psychosocial distress such as psychiatric disorders, depression and suicidality [28] that may not be completely reversible [13]. As shown in Table 2, normal-weight adolescents who perceive themselves as overweight have the highest probability of using unsafe weight-loss methods among all subgroups.

### 2.4. Covariate Variables

To control for confounding factors, we not only conduct analyses separately for four subsamples based on gender and the reported weight status, but also use a total of 16 covariate variables in addition to regional and year dummies. Specifically, we include the BMI category and age as they are significant predictors of weight perceptions and are expected to be correlated with weight-loss behaviors. We include three dummy variables for race and ethnicity (Non-Hispanic white, Hispanic, and African Americans). We include grade indicators to capture the effect of peer pressure on body image in school. The average GPA, which is a proxy for cognitive ability and the awareness of nutrition and physical activity, is also included. We also include two variables for risk behaviors and factors indicating the history of smoking, drinking, and drug usage, driving under influence, sexual behaviors, and suicide attempts and plans.

## 3. Estimation Strategy

Let *Y*_1_(*Y*_0_) be weight-loss intentions or behaviors when an adolescent perceives himself/herself as overweight (non-overweight). The treatment (control) group consists of adolescents who (do not) perceive themselves as overweight. The treatment effect of overweight perception is the difference between two outcomes: *Y*_1_ – *Y*_1_. However, this difference is not observable due to a missing data problem: perceived overweight (non-overweight) reveals *Y*_1_(*Y*_0_), but conceals the other potential outcome. Since overweight perception is likely to be endogenous and lack of random assignment of self-perceived overweight status causes selection bias, we employ propensity score matching (PSM) to address selection bias. The details of PSM can be found at Abadie and Imbens (2002), Hahn (1998), Heckman *et al.*, (1998), and Hirano *et al.*, (2003) [29,30,31,32]. The PSM has two critical assumptions:

A1. Conditional Independence Assumption: (*Y*_0_ − *Y*_1_)*T*|*X* (*Y*_0_, *Y*_1_)⊥*T*|*X*; and
A2. Common Support Assumption: 0 < *prob*(*T* = 1|*X*) < 1

Where ⊥ is the notation for statistical independence and *T* indicates the treatment status. Assumption A1 says that all the variables driving self-selection are observable to researchers and the treatment assignment is independent of outcomes conditional on covariates. Assumption A2 says that the probability of participation in treatment is bounded between zero and one. Based on these two assumptions, the estimated counterfactual outcome of treated individual *i* is:

(1) $${\hat{Y}}_{0i}=\underset{j\in C_{i}^{0}}{\sum }\left(w_{ij}Y_{j}|T_{j}=0\right)$$

where $C_{i}^{0}$ is the set of matches of individual *i*, *w_ij_* ∈ [0,1] is the weight of matched counterfactual outcomes, and ∑*w_ij_* = 1. Since we are interested in knowing whether self-perception of being overweight has any effect on adolescents’ weight-loss efforts, we focus on the sample average treatment effect on the treated (SATT):

(2) $$SATT=\frac{1}{N}\underset{i|T_{i}=1}{\sum }\left(Y_{1i}-{\hat{Y}}_{0i}\right)$$

where *N*_1_ = ∑_*i*_ *Y_i_* and *Y*_0_ is the estimated potential outcome if not treated in Equation (1).

We employ two widely used PSM matching algorithms: nearest neighbor matching (NNM) and local linear matching (LLM). The NNM estimator compares every treated unit with one or more units from the comparison group that are closest in terms of the propensity score. It defines the set of matches with replacement is given below:

(3) $$C_{i}^{0}\left(M\right)=\left\{l=1,\cdots ,N\left|T_{i}=0,|P_{i}-P_{j}\right|<d_{i}\left(M\right)\right\}.$$

where *M* indicates the number of matches (neighbors) and *d_i_*(*M*) is the distance from individual *i* to the *M^th^* nearest match in the comparison group. We implicitly define *d_i_*(*M*) as follows:

(4) $$\sum_{l:\;T_{l=0}}1\left\{|P_{i}-P_{j}|<d_{i}\left(M\right)\right\}<M$$

and:

(5) $$\underset{l:\;T_{l=0}}{\sum }1\left\{|P_{i}-P_{j}|<d_{i}\left(M\right)\right\}\geq M$$

where 1(⋅) is the indicator function, which equals to 1 when the value in brackets is true, and zero otherwise. We implement this method using one or five nearest neighbors and with replacement. We impose the common support restriction and each match is weighted equally.

The LLM uses a kernel-weighted average over multiple persons in the comparison group as the counterfactual outcome of the treated observation. Fan (1992) shows that LLM converges faster and that it is more robust to different densities of data than kernel matching [33]. The weight of LLM is given by the following:

(6) $$w_{ij}=\frac{G_{ij}\sum_{l\in C_{i}^{0}}{\left(P_{i}-P_{j}\right)}^{2}-\left[G_{ij}\left(P_{i}-P_{j}\right)\right]\left\{\sum_{l\in C_{i}^{0}}\left(P_{i}-P_{j}\right)\right\}}{\sum_{j\in C_{i}^{0}}\left[G_{ij}\sum_{l\in C_{i}^{0}}G_{il}{\left(P_{i}-P_{j}\right)}^{2}\right]-{\left[\sum_{l\in C_{i}^{0}}G_{il}\left(P_{i}-P_{j}\right)\right]}^{2}}$$

where *G_ij_* = *G*((*P_j_* − *P_i_*)/*h*), and *h* is the bandwidth. We use the Epanechnikov distribution as the kernel function.

To test the hypothesis, we need to estimate the standard errors for the estimators. However, calculating analytical standard errors can be cumbersome. Bootstraping is often used to obtain standard errors for matching estimators (see details at Black and Smith, Heckman, *et al.*, Sianesi [34,35,36]). Each bootstrap sample is a random sampling with replacement from the original data set. We draw 500 bootstrap samples and estimate 500 average treatment effects for the treated. The distribution of these means approximates the sampling distribution (and thus the standard error) of the population mean.

Researchers have documented the following potential advantages of matching technique. First, matching does not impose any specific functional form between the dependent variable and independent variables, thus avoiding possible model misspecification errors [37]. The so-called LaLonde’s critiques suggest that non-experimental estimates are sensitive to model specification and differ greatly from the experimental estimates [38]. Second, matching can impose a common support requirement. The poor overlap on support between the treated and untreated groups raises questions about the robustness of parametric methods relying on the functional form to extrapolate outside the common support [39,40]. Third, matching allows endogenous covariates [41]. Although matching techniques have advantages over other non-experimental evaluation techniques when lacking exogenous changes in weight perception, challenges still exist. First, matching techniques require a large number of observations and a rich set of covariates. It is less a problem for this study due to large sample size (more than 50,000 observations) and a rich set of information collected for each respondent (see details in Section 3). In addition to region and year dummies, we incorporate 16 covariates as matching variables. All of the matching variables are not only correlated with weight perceptions and weight-loss intentions and behaviors, but also demonstrate substantial overlaps between the treatment and comparison groups as suggested by the distribution of the BMI percentile between two groups (see Appendix B). Second, matching techniques assume that all the variables driving self-selection are observable to researchers, *i.e.*, the treatment assignment is independent of outcomes conditional on covariates [38]. We divide the sample by gender to eliminate the gender differences of weight perceptions and behaviors and by weight status based on the reported weight and height to eliminate the unobserved differences between normal-weight and overweight adolescents. We also conduct a series of robustness analyses using covariate matching and the bivariate probit model with endogenous self-perception of being overweight.

## 4. Results and Discussions

### 4.1. Main Matching Results and Discussions

For all estimations we impose common support to ensure that characteristics observed in the treatment group is also observed in the comparison group. Since trimming could theoretically improve the matching quality and reduce the bias [35], we trim the sample by one percent. Table 3 summarizes the estimated treatment effects for the treated based on the nearest neighbor matching (NNM) with five neighbors and the local linear regression matching (LLR) with a plug-in rule-of-thumb bandwidth. Every estimate is a percentage point (not a percent change) representing the difference in the probability of certain outcomes between an average adolescent with and without self-perception of being overweight. For example, if the probability of exercising to lose weight is 20% for the self-perceived normal-weight group, an increase of four percentage points from 20% to 24% translates to the treatment effect of 0.04 which is equivalent to a 20 percent increase. The results based on the NNM and LLR algorithms are remarkably similar. This is exactly what the theory predicts: when the sample size is large enough all matching algorithms produce the same results [42]. We summarize the findings separately for normal-weight and overweight adolescents.

As show in Columns 1–4 of Panel A in Table 3, compared with overweight adolescents who do not perceive themselves as overweight, overweight adolescents with a correct perception have a much stronger intention to lose weight in general or through diet or exercise. For example, self-perception of being overweight increases the general weight-loss intention by approximately 20 percentage points for females and 37 percentage points for males. However, a correct self-perception of being overweight is not found to be associated with improvements in eating habits. The probability of meeting the recommended consumption levels of fruits and vegetables among overweight adolescents with a correct weight perception is five percentage points lower for males and two percentage points lower for females, respectively; and none of the estimates on soft drink consumption is statistically significant (see Columns 1–4 of Panel B in Table 3). On the other hand, a correct perception of own weight status does not have any statistically significant effect on exercises behaviors among overweight female adolescents (see Columns 1–2 in Panel C of Table 3). Overweight male adolescents with a correct perception of their weight status are found to be less physically active, less likely to engage in moderate or vigorous exercise, and less likely to attend PE classes or play on team sports than their counterparts (see Columns 3–4 in Panel C of Table 3). To summarize, among overweight adolescents, self-perception of being overweight enhances intention to lose weight but fail to improve eating and exercise behaviors, which is similar as the findings for U.S. adults by Fan and Jin [43]. Instead, self-perception of being overweight increases the probability of engaging in unsafe and health-compromising weight-loss strategies.

For the normal-weight subsample, self-perception of being overweight increases weight-loss intentions as well as the probability of engaging in unsafe and health-compromising weight-loss strategies (Columns 5–8 in Panels A and D of Table 3). The effects of the self-perception of being overweight on weight loss intentions for the normal-weight subsample are almost twice as large as those for the overweight subsample. Overweight perception leads to higher probability (approximately 6–11 percentage points) of using extreme weight-loss methods among normal-weight adolescent, while its effects are much smaller and less statistically significant for overweight adolescents. The findings indicate that normal-weight adolescents who perceive themselves as overweight are more obsessed with body image and have a much stronger intention to lose weight. However, they do not engage in healthy eating and become physical active. The probability of meeting the recommended consumption levels of fruits and vegetables as well as soda consumption is not statistically different between normal-weight adolescents with and without overweight perception (Columns 5–8 in Panel B of Table 3). If normal-weight adolescents mistakenly perceive themselves as being overweight, they are less likely to be physically active, engage in moderate or vigorous exercise, attend PE classes, and play on team sports; instead, they watch more TV and play more video games (Columns 7–8 in Panel C of Table 3). The results suggest that weight misperception among normal-weight adolescents could exacerbate eating disorders and other adverse health consequences as they favor extreme weight-loss methods to gain immediate effects. Our findings highlight the importance of having a correct perception of weight status even for normal-weight adolescents.

The results show that having a correct self-perception of weight status reduces the possibility of taking extreme weight-loss methods among normal-weight adolescents and increases weight-loss intentions among overweight adolescents. More important, the results show a gap between weight-loss intentions and behaviors. The gap can be explained by time-inconsistent preferences [44,45]. Hyperbolic individuals are more likely to choose immediate rewards/gratification and encounter greater difficulties in delaying gratification [46,47], which leads to overconsumption of food, especially excessively cheap and unhealthy food [48]. They also greatly discount the long-run benefits of nutritious meals and exercise [47,49]. Fan and Jin (2013) find that overweight and obese adults have a lower self-control than normal-weight individuals in the United States; and individuals who are lack of self-control are more likely to have poor eating and exercise habits [43]. Normal-weight adolescents with self-perception of being overweight might be impatient and hyperbolic discounting leads them to use extreme weight-loss strategies with immediate effects and procrastinate adopting healthy weight-loss strategies such as healthy eating and being physically active.

**Table 3:** Effects of the Self-perception of Being Overweight on Weight-loss Intentions and Behaviors using Propensity Score Matching.

	Self-Reported Overweight Sample				Self-Reported Normal-weight Sample			
	Female		Male		Female		Male	
	NNM	LLR	NNM	LLR	NNM	LLR	NNM	LLR
	(1)	(2)	(3)	(4)	(5)	(6)	(7)	(8)
	**Panel A: Weight-loss Intentions**							
LoseWeight	0.188 **	0.203 **	0.366 **	0.366 **	0.33 **	0.313 **	0.539 **	0.551 **
	(0.018)	(0.016)	(0.017)	(0.014)	(0.01)	(0.008)	(0.018)	(0.014)
LoseWight_Diet	0.148 **	0.145 **	0.218 **	0.212 **	0.235 **	0.219 **	0.313 **	0.309 **
	(0.02)	(0.017)	(0.017)	(0.014)	(0.011)	(0.009)	(0.019)	(0.016)
LoseWeight_Exer	0.075 **	0.082 **	0.123 **	0.117 **	0.12 **	0.104 **	0.245 **	0.227 **
	(0.018)	(0.016)	(0.016)	(0.013)	(0.011)	(0.008)	(0.019)	(0.015)
	**Panel B: Eating Habits**							
Fruit_2	0.003	−0.01	−0.057 **	−0.05 **	−0.015	−0.019*	−0.002	−0.011
	(0.016)	(0.014)	(0.014)	(0.012)	(0.009)	(0.008)	(0.015)	(0.012)
Vegetable_3	−0.007	−0.01	−0.026 **	−0.023 **	−0.002	−0.002	−0.012	−0.002
	(0.011)	(0.008)	(0.009)	(0.007)	(0.005)	(0.004)	(0.008)	(0.007)
Soda	−0.042	−0.016	−0.024	−0.036	−0.013	−0.017	0.011	0.024
	(0.034)	(0.027)	(0.026)	(0.023)	(0.016)	(0.014)	(0.03)	(0.024)
	**Panel C: Physical and Sedentary Activity**							
Active_5 Days	−0.028	−0.036	−0.101 **	−0.116 **	−0.031 *	−0.04 **	−0.122 **	−0.12 **
	(0.026)	(0.021)	(0.023)	(0.018)	(0.014)	(0.011)	(0.025)	(0.02)
Moderate_Exer	0.002	−0.008	−0.061 **	−0.065 **	−0.011	−0.017	−0.102 **	−0.105 **
	(0.022)	(0.018)	(0.015)	(0.012)	(0.012)	(0.01)	(0.019)	(0.015)
Vigorous_Exer	−0.006	0.002	−0.049 **	−0.051 **	−0.015	−0.008	−0.053 **	−0.052 **
	(0.019)	(0.015)	(0.017)	(0.013)	(0.01)	(0.009)	(0.019)	(0.014)
PE_Class	0.006	−0.011	−0.073 **	−0.07 **	−0.033 **	−0.034 **	−0.077 **	−0.08 **
	(0.022)	(0.018)	(0.017)	(0.014)	(0.013)	(0.01)	(0.019)	(0.015)
Sport_Team	−0.024	−0.018	−0.107 **	−0.115 **	−0.022	−0.028 **	−0.131 **	−0.139 **
	(0.023)	(0.019)	(0.017)	(0.014)	(0.012)	(0.01)	(0.02)	(0.016)
TV_Time	0.007	0.011	0.04 *	0.032 *	−0.022	−0.016	0.039	0.033 *
	(0.022)	(0.018)	(0.018)	(0.015)	(0.012)	(0.009)	(0.02)	(0.016)
Video_Time	0.015	0.011	0.002	0.01	−0.001	0.002	0.063 **	0.05 **
	(0.022)	(0.017)	(0.019)	(0.016)	(0.011)	(0.009)	(0.02)	(0.016)
	**Panel D: Unhealthy Weight-loss Methods**							
LoseWeight_Fasting	0.01	0.019	0.019	0.024 **	0.11 **	0.109 **	0.09 **	0.087 **
	(0.019)	(0.015)	(0.011)	(0.009)	(0.01)	(0.008)	(0.012)	(0.01)
LoseWeight_Med	0.038*	0.051 **	0.018	0.022 **	0.095 **	0.098 **	0.063 **	0.061 **
	(0.017)	(0.013)	(0.01)	(0.008)	(0.008)	(0.007)	(0.011)	(0.009)

Bootstrapped standard errors are in parentheses. Asterisks, ** and *, indicate the 1%, and 5% significance level, respectively.

### 4.2. Quality of Matching

**Figure 2:**
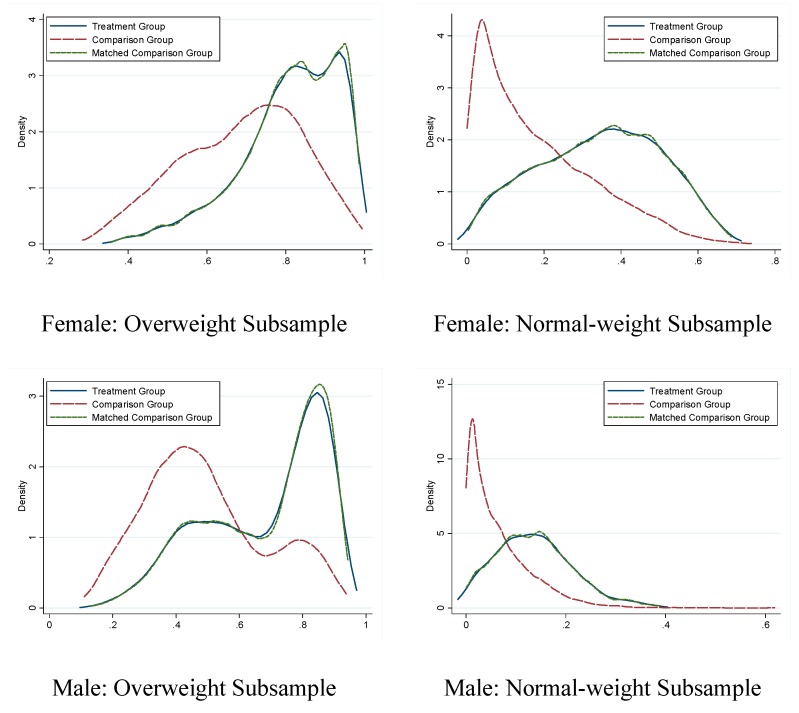
Kernel Density Estimate of the Distribution of the Propensity Score based on Nearest Neighbor Matching.

To check the quality of matching we conduct balancing tests of matching covariates and compare the propensity score between the treatment and comparison groups before and after matching for each outcome variable. Take the general weight-loss intention as an example. As shown in Table 4, out of 16 reported matching covariates, 14 for the normal-weight subsample and 12 for the overweight subsample are statistically significant at the 5% level before matching for females, and the corresponding numbers for males are 8 and 10. After matching, none of the differences between two groups are still statistically significant and the magnitude of the differences also decreases dramatically. Figure 2 shows the distributions of the propensity scores before and after matching for all four subsamples by gender and self-reported weight status. It is clear that the propensity score destitutions differ significantly between the treatment and comparison groups before matching, but they almost completely overlap after matching. We conclude that matching indeed is effective in eliminating the differences between the treatment and control groups. The matching quality is similar for other dependent variables.

### 4.3. Robustness Checks

We provide five robustness checks for the main results. First, we experiment with different PSM specifications. In particular, we incorporate other risk behavior factors into the propensity score function. Those binary risk variables indicate whether the respondent had suicidal thoughts during last 12 months; always wore a seatbelt while driving or being a passenger in a car; had a physical fight during last 12 months; ever smoked, drank, used cocaine, or used any other drug during past 30 days, respectively; had sexual intercourse with more than 4 people; and had sex during the past three months. We also employ different matching parameters—using one or ten neighbors in the comparison group to match every treated individual for the NNM estimator and a series of fixed bandwidths for the LLM estimator. We also match without trimming. The treatment effects based on each of the new specifications are very similar to the main results (see Table 5, Table 6 and Table 7).

**Table 4:** Balancing Tests of Matching Covariates for the Outcome Variable (*LoseWeight*) ^a^.

		Female				Male			
U = Unmatched		Self-Reported Normal-Weight		Self-Reported Overweight		Self-Reported Normal-Weight		Self-Reported Overweight	
M = Matched		Difference	*p ^b^*	Difference	*p ^b^*	Differenc	*p ^b^*	Difference	*p ^b^*
BMI category	U	1.860	0.000	0.250	0.000	1.752	0.000	0.380	0.000
	M	0.051	0.176	0.007	0.438	0.036	0.612	−0.002	0.824
Age (years)	U	0.102	0.000	0.244	0.000	0.042	0.259	0.028	0.310
	M	−0.003	0.920	−0.025	0.304	0.016	0.755	0.009	0.737
Non-Hispanic White	U	0.041	0.000	0.162	0.000	−0.014	0.368	0.071	0.000
	M	0.010	0.354	0.009	0.354	0.006	0.762	0.014	0.174
Hispanics	U	0.043	0.000	0.018	0.181	0.043	0.001	0.028	0.008
	M	−0.012	0.241	−0.008	0.391	−0.008	0.671	−0.018	0.055
African American	U	−0.094	0.000	−0.194	0.000	−0.073	0.000	−0.106	0.000
	M	0.002	0.782	0.009	0.243	0.003	0.793	0.006	0.352
GPA	U	0.157	0.000	0.122	0.000	0.242	0.000	0.280	0.000
	M	−0.012	0.556	0.028	0.136	−0.017	0.684	0.001	0.956
10th grade	U	0.000	0.985	−0.037	0.004	−0.016	0.222	−0.013	0.200
	M	0.000	0.980	−0.005	0.546	0.008	0.630	−0.004	0.647
11th grade	U	0.013	0.085	0.038	0.003	−0.001	0.951	0.003	0.784
	M	0.005	0.597	0.008	0.345	0.004	0.847	0.006	0.494
12th grade	U	0.019	0.015	0.078	0.000	0.021	0.121	0.019	0.065
	M	−0.001	0.902	−0.007	0.425	−0.003	0.882	0.001	0.884
Depression	U	0.088	0.000	0.050	0.001	0.069	0.000	0.030	0.002
	M	−0.021	0.060	0.004	0.684	−0.013	0.491	−0.009	0.290
Suicide	U	0.066	0.000	0.057	0.000	0.058	0.000	0.022	0.003
	M	−0.013	0.148	0.005	0.580	−0.005	0.747	−0.006	0.340
Sex	U	−0.018	0.038	−0.126	0.000	−0.081	0.000	−0.142	0.000
	M	0.003	0.818	0.007	0.501	0.000	0.993	−0.012	0.245
Drink	U	0.053	0.000	0.019	0.121	0.004	0.801	−0.033	0.002
	M	−0.005	0.624	−0.008	0.343	0.000	0.993	−0.016	0.102
Smoke	U	0.059	0.000	0.006	0.659	−0.024	0.113	−0.005	0.663
	M	−0.010	0.375	0.001	0.927	−0.002	0.919	−0.012	0.232
Marijuana	U	0.038	0.000	−0.051	0.000	−0.048	0.002	−0.034	0.003
	M	−0.002	0.850	0.000	0.962	0.005	0.812	−0.006	0.548
Drive	U	0.033	0.000	−0.019	0.160	−0.005	0.755	−0.016	0.137
	M	−0.008	0.446	−0.005	0.614	0.003	0.865	−0.007	0.476

^a^ All tests for the mean difference of each matching covariate between the comparison and treatment groups are based on the PSM with five neighbors. Let *n_1_* and *n_2_* represent the number of observations in the treatment and comparison groups on the support. The corresponding t-statistics are calculated as $\left({\bar{X}}_{treat}-{\bar{X}}_{control}\right)=\left({\bar{X}}_{treat}-{\bar{X}}_{control}\right)/\sqrt{\frac{\sigma_{treat}^{2}}{n_{1}}+\frac{\sigma_{control}^{2}}{n_{1}}}$. Figures in bold indicate the mean difference is statistically different at the 10% significance level; ^b^ *P*-value for the equal mean of each matching covariate between the treatment and comparison groups.

**Table 5:** Propensity Score Matching Results using an Expanded Set of Risk Behavior Variables.

	Self-Reported Overweight Sample				Self-Reported Normal-weight Sample			
	Female		Male		Female		Male	
	NNM	LLR	NNM	LLR	NNM	LLR	NNM	LLR
	**Panel A: Weight-loss Intentions**							
LoseWeight	0.192 **	0.204 **	0.345 **	0.358 **	0.326 **	0.303 **	0.564 **	0.554 **
	(0.019)	(0.017)	(0.017)	(0.014)	(0.011)	(0.008)	(0.020)	(0.017)
LoseWight_Diet	0.142 **	0.142 **	0.224 **	0.217 **	0.227 **	0.22 **	0.308 **	0.306 **
	(0.024)	(0.019)	(0.020)	(0.018)	(0.012)	(0.010)	(0.020)	(0.017)
LoseWeight_Exer	0.068 **	0.080 **	0.120 **	0.118 **	0.115 **	0.102 **	0.239 **	0.231 **
	(0.02)	(0.017)	(0.018)	(0.013)	(0.011)	(0.009)	(0.022)	(0.015)
	**Panel B: Eating Habits**							
Fruit_2	0.001	−0.002	−0.053 **	−0.052 **	−0.006	−0.011	−0.017	−0.012
	(0.019)	(0.015)	(0.015)	(0.013)	(0.011)	(0.008)	(0.016)	(0.013)
Vegetable_3	−0.015	−0.009	−0.025 *	−0.023 **	−0.003	−0.001	0.005	0.003
	(0.011)	(0.008)	(0.010)	(0.008)	(0.005)	(0.004)	(0.009)	(0.007)
Soda	−0.040	−0.007	−0.029	−0.034	−0.031	−0.021	0.016	0.032
	(0.034)	(0.031)	(0.027)	(0.025)	(0.018)	(0.015)	(0.027)	(0.022)
	**Panel C: Physical and Sedentary Activity**							
Active_5 Days	−0.041	−0.041	−0.089 **	−0.104 **	−0.032 *	−0.038 **	−0.112 **	−0.116 **
	(0.025)	(0.022)	(0.022)	(0.017)	(0.016)	(0.013)	(0.027)	(0.022)
Moderate_Exer	0.002	−0.018	−0.049 **	−0.064 **	−0.002	−0.017	−0.109 **	−0.103 **
	(0.024)	(0.020)	(0.019)	(0.016)	(0.012)	(0.010)	(0.019)	(0.017)
Vigorous_Exer	−0.017	−0.010	−0.047 *	−0.049 **	−0.010	−0.008	−0.056 **	−0.054 **
	(0.02)	(0.016)	(0.019)	(0.015)	(0.012)	(0.009)	(0.021)	(0.017)
PE_Class	0.008	−0.014	−0.096 **	−0.076 **	−0.020	−0.026 *	−0.080 **	−0.085 **
	(0.023)	(0.016)	(0.019)	(0.016)	(0.016)	(0.011)	(0.022)	(0.015)
Sport_Team	−0.032	−0.026	−0.103 **	−0.112 **	−0.020	−0.023 *	−0.122 **	−0.135 **
	(0.02)	(0.017)	(0.019)	(0.015)	(0.013)	(0.011)	(0.020)	(0.015)
TV_Time	0.004	0.015	0.033	0.034 *	−0.014	−0.012	0.054 **	0.047 **
	(0.023)	(0.02)	(0.018)	(0.014)	(0.012)	(0.009)	(0.02)	(0.016)
Video_Time	−0.011	0.001	−0.004	0.005	−0.005	0.000	0.034	0.046 **
	(0.026)	(0.017)	(0.02)	(0.015)	(0.011)	(0.008)	(0.021)	(0.018)
	**Panel D: Unhealthy Weight-loss Methods**							
LoseWeight_Fasting	0.026	0.033 *	0.023 *	0.025 *	0.109 **	0.113 **	0.080 **	0.080 **
	(0.019)	(0.015)	(0.011)	(0.010)	(0.01)	(0.008)	(0.012)	(0.010)
LoseWeight_Med	0.029	0.042 **	0.022 *	0.024 **	0.099 **	0.099 **	0.054 **	0.056 **
	(0.019)	(0.013)	(0.01)	(0.008)	(0.009)	(0.008)	(0.011)	(0.009)

Bootstrapped standard errors are in parentheses. Asterisks, ** and *, indicate the 1%, and 5% significance level, respectively. NNM uses 5 neighbors in the comparison group and LLR uses a rule-of-thumb bandwidth.

**Table 6:** Propensity Score Matching Results using One Neighbor and a Fixed Bandwidth.

	Self-Reported Overweight Sample				Self-Reported Normal-weight Sample				
	Female		Male		Female			Male	
	NNM	LLR	NNM	LLR	NNM	LLR	NNM		LLR
	**Panel A: Weight-loss Intentions**								
LoseWeight	0.181 **	0.187 **	0.374 **	0.36 **	0.34 **	0.329 **	0.555 **		0.550 **
	(0.020)	(0.018)	(0.018)	(0.014)	(0.012)	(0.007)	(0.023)		(0.014)
LoseWight_Diet	0.143 **	0.145 **	0.199 **	0.208 **	0.241 **	0.231 **	0.314 **		0.313 **
	(0.024)	(0.02)	(0.02)	(0.014)	(0.015)	(0.01)	(0.023)		−00.016
LoseWeight_Exer	0.091 **	0.078 **	0.120 **	0.120 **	0.118 **	0.121 **	0.265 **		0.241 **
	(0.019)	(0.019)	(0.02)	(0.013)	(0.014)	(0.009)	(0.026)		(0.014)
	**Panel B: Eating Habits**								
Fruit_2	−0.005	−0.004	−0.05 **	−0.047 **	−0.013	−0.017 *	−0.009		−0.008
	(0.017)	(0.015)	(0.017)	(0.012)	(0.012)	(0.008)	(0.023)		(0.015)
Vegetable_3	−0.011	−0.013	−0.025 *	−0.028 **	−0.009	−0.003	−0.009		−0.002
	(0.012)	(0.011)	(0.010)	(0.009)	(0.006)	(0.004)	(0.010)		(0.006)
Soda	−0.054	−0.036	−0.032	−0.028	−0.005	−0.025	−0.037		0.015
	(0.038)	(0.032)	(0.033)	(0.025)	(0.022)	(0.016)	(0.035)		(0.025)
	**Panel C: Physical and Sedentary Activity**								
Active_5Days	−0.025	−0.022	−0.089 **	−0.095 **	−0.031	−0.033 **	−0.100 **		−0.110 **
	(0.028)	(0.021)	(0.024)	(0.020)	(0.020)	(0.012)	(0.038)		(0.021)
Moderate_Exer	−0.002	0.010	−0.057 **	−0.061 **	−0.012	−0.011	−0.095 **		−0.097 **
	(0.026)	(0.020)	(0.017)	(0.013)	(0.017)	(0.011)	(0.023)		(0.014)
Vigorous_Exer	−0.010	−0.006	−0.044*	−0.05 **	−0.014	−0.006	−0.054 *		−0.049 **
	(0.021)	(0.018)	(0.020)	(0.013)	(0.015)	(0.010)	(0.026)		(0.014)
PE_Class	−0.004	00.00	−0.058 **	−0.084 **	−0.018	−0.031 **	−0.076 **		−0.074 **
	(0.024)	(0.022)	(0.019)	(0.016)	(0.015)	(0.009)	(0.028)		(0.016)
Sport_Team	−0.030	−0.010	−0.121 **	−0.109 **	−0.017	−0.022 *	−0.143 **		−0.126 **
	(0.028)	(0.024)	(0.020)	(0.016)	(0.015)	(0.01)	(0.025)		(0.015)
TV_Time	0.009	0.011	0.0400	0.031	−0.026	−0.022 *	0.029		0.029
	(0.022)	(0.018)	(0.022)	(0.016)	(0.016)	(0.010)	(0.028)		(0.016)
Video_Time	0.007	0.006	−0.002	0.002	0.005	0.000	0.068 **		0.046 **
	(0.027)	(0.023)	(0.024)	(0.019)	(0.014)	(0.009)	(0.025)		(0.016)
	**Panel D: Unhealthy Weight-loss Methods**								
LoseWeight_Fasting	0.010	0.003	0.026 *	0.021	0.105 **	0.109 **	0.087 **		0.086 **
	(0.023)	(0.019)	(0.013)	(0.011)	(0.012)	(0.008)	(0.015)		0.011
LoseWeight_Med	0.021	0.036 *	0.017	0.019 *	0.091 **	0.097 **	0.071 **		0.063 **
	(0.017)	(0.018)	(0.012)	(0.010)	(0.011)	(0.008)	(0.012)		(0.009)

Bootstrapped standard errors are in parentheses. Asterisks, ** and *, indicate the 1%, and 5% significance level, respectively.

**Table 7:** Propensity Score Matching Results without Trimming.

	Self-Reported Overweight Sample				Self-Reported Normal-weight Sample			
	Female		Male		Female		Male	
	NNM	LLR	NNM	LLR	NNM	LLR	NNM	LLR
	**Panel A: Weight-loss Intentions**							
LoseWeight	0.189 **	0.186 **	0.366 **	0.360 **	0.329 **	0.328 **	0.539 **	0.550 **
	(0.019)	(0.017)	(0.015)	(0.014)	(0.01)	(0.007)	(0.018)	(0.014)
LoseWight_Diet	0.148 **	0.146 **	0.218 **	0.208 **	0.235 **	0.231 **	0.313 **	0.312 **
	(0.020)	(0.020)	(0.016)	(0.015)	(0.011)	(0.01)	(0.018)	(0.017)
LoseWeight_Exer	0.075 **	0.077 **	0.125 **	0.120 **	0.120 **	0.120 **	0.243 **	0.241 **
	(0.018)	(0.018)	(0.016)	(0.013)	(0.011)	(0.009)	(0.018)	(0.014)
	**Panel B: Eating Habits**							
Fruit_2	0.002	−0.005	−0.058 **	−0.047 **	−0.015	−0.017 *	−0.004	−0.009
	(0.015)	(0.016)	(0.013)	(0.012)	(0.009)	(0.008)	(0.016)	(0.015)
Vegetable_3	−0.008	−0.012	−0.026 **	−0.028 **	−0.002	−0.002	−0.012	−0.002
	(0.011)	(0.011)	(0.008)	(0.009)	(0.005)	(0.004)	(0.007)	(0.006)
Soda	−0.041	−0.036	−0.024	−0.029	−0.012	−0.025	0.010	0.016
	(0.030)	(0.030)	(0.026)	(0.024)	(0.017)	(0.016)	(0.027)	(0.024)
	**Panel C: Physical Activity**							
Active_5 Days	−0.025	−0.021	−0.099 **	−0.094 **	−0.030 *	−0.032 **	−0.122 **	−0.106 **
	(0.023)	(0.021)	(0.023)	(0.02)	(0.014)	(0.012)	(0.028)	(0.021)
Moderate_Exer	0.000	0.011	−0.06 **	−0.062 **	−0.011	−0.011	−0.100 **	−0.095 **
	(0.020)	(0.020)	(0.013)	(0.013)	(0.013)	(0.011)	(0.018)	(0.014)
Vigorous_Exer	−0.004	−0.006	−0.048 **	−0.049 **	−0.014	−0.005	−0.054 **	−0.05 **
	(0.018)	(0.018)	(0.017)	(0.014)	(0.012)	(0.010)	(0.020)	(0.014)
PE_Class	0.004	−0.001	−0.072 **	−0.083 **	−0.035 **	−0.032 **	−0.079 **	−0.076 **
	(0.021)	(0.022)	(0.017)	(0.016)	(0.011)	(0.009)	(0.019)	(0.016)
Sport_Team	−0.021	−0.009	−0.106 **	−0.109 **	−0.024 *	−0.022 *	−0.132 **	−0.127 **
	(0.026)	(0.024)	(0.017)	(0.016)	(0.011)	(0.01)	(0.019)	(0.015)
TV_Time	0.007	0.012	0.039 *	0.030	−0.023 *	−0.023 *	0.037	0.027
	(0.02)	(0.018)	(0.018)	(0.016)	(0.012)	(0.010)	(0.020)	(0.017)
Video_Time	0.015	0.005	0.001	0.002	−0.001	0.000	0.062 **	0.045 **
	(0.025)	(0.023)	(0.020)	(0.018)	(0.011)	(0.009)	(0.02)	(0.016)
	**Panel D: Unhealthy Weight-loss Methods**							
LoseWeight_Fasting	0.010	0.003	0.019	0.021	0.109 **	0.108 **	0.089 **	0.085 **
	(0.021)	(0.019)	(0.012)	(0.011)	(0.009)	(0.008)	(0.013)	(0.011)
LoseWeight_Med	0.036*	0.035	0.018	0.018	0.095 **	0.098 **	0.063 **	0.063 **
	(0.018)	(0.018)	(0.010)	(0.010)	(0.009)	(0.008)	(0.010)	(0.009)

Notes: Bootstrapped standard errors are in parentheses. Asterisks, ** and *, indicate the 1%, and 5% significance level, respectively. NNM uses 5 neighbors in the comparison group and LLR uses a fixed bandwidth (0.1).

Second, we try a different matching mechanism, covariate matching (CVM) with the same set of covariates as the main specification. The main difference between the PSM and the CVM lies on the imputation of the missing potential outcomes. The PSM uses the estimated propensity score and the CVM uses untreated individuals with similar values of covariates (see Abadie, *et al.*, (2004) for a detailed discussion for the CVM [50]). The results from the CVM are remarkably consistent with the main results (see Table 8).

**Table 8:** Effects of the Self-perception of Being Overweight on Weight-loss Intentions and Behaviors using Covariate Matching.

	Self-Reported Overweight Sample				Self-Reported Normal-Weight Sample			
	Female		Male		Female			Male
	*N* = 5	*N* = 10	*N* = 5	*N* = 10	*N* = 5	*N* = 10	*N* = 5	*N* = 10
	**Panel A: Weight-loss Intentions**							
LoseWeight	0.195 **	0.195 **	0.355 **	0.357 **	0.316 **	0.317 **	0.555 **	0.558 **
	(0.015)	(0.014)	(0.012)	(0.011)	(0.007)	(0.007)	(0.014)	−00.014
LoseWight_Diet	0.143 **	0.144 **	0.222 **	0.218 **	0.222 **	0.222 **	0.300 **	0.306 **
	(0.016)	(0.016)	(0.012)	(0.012)	(0.009)	(0.008)	(0.016)	−00.016
LoseWeight_Exer	0.090 **	0.088 **	0.118 **	0.119 **	0.108 **	0.105 **	0.231 **	0.231 **
	(0.015)	(0.015)	(0.012)	(0.011)	(0.008)	(0.008)	(0.015)	−00.014
	**Panel B: Eating Habits**							
Fruit_2	0.007	0.001	−0.039 **	−0.040 **	−0.018 *	−0.016 *	−0.007	−00.004
	(0.012)	(0.012)	(0.01)	(0.01)	(0.007)	(0.007)	(0.012)	−00.012
Vegetable_3	−0.004	−0.007	−0.019 **	−0.019 **	0.002	00.00	00.00	00.002
	(0.007)	(0.007)	(0.005)	(0.005)	(0.004)	(0.004)	(0.007)	−00.006
Soda	−0.028	−0.020	−0.031	−0.033	−0.019	−0.020	0.006	00.013
	(0.023)	(0.022)	(0.020)	(0.019)	(0.013)	(0.013)	(0.025)	−00.024
	**Panel C: Physical and Sedentary Activity**							
Active_5Days	−0.020	−0.023	−0.109 **	−0.113 **	−0.033 **	−0.039 **	−0.109 **	−0.114 **
	(0.019)	(0.018)	(0.017)	(0.016)	(0.012)	(0.011)	(0.021)	−00.02
Moderate_Exer	0.007	0.006	−0.057 **	−0.06 **	−0.016	−0.016	−0.102 **	−0.102 **
	(0.016)	(0.016)	(0.011)	(0.011)	(0.01)	(0.009)	(0.016)	−00.015
Vigorous_Exer	0.001	0.001	−0.044 **	−0.047 **	−0.009	−0.006	−0.051 **	−0.051 **
	(0.014)	(0.013)	(0.012)	(0.011)	(0.008)	(0.008)	(0.015)	−00.014
PE_Class	−0.012	−0.014	−0.08 **	−0.078 **	−0.03 **	−0.025 **	−0.072 **	−0.069 **
	(0.015)	(0.015)	(0.012)	(0.012)	(0.009)	(0.009)	(0.016)	−00.015
Sport_Team	−0.013	−0.014	−0.107 **	−0.110 **	−0.025 **	−0.027 **	−0.130 **	−0.137 **
	(0.016)	(0.016)	(0.012)	(0.012)	(0.01)	(0.009)	(0.017)	−00.016
TV_Time	0.010	0.012	0.043 **	0.046 **	−0.005	−0.006	0.040*	0.045 **
	(0.016)	(0.016)	(0.012)	(0.012)	(0.009)	(0.009)	(0.016)	−00.015
Video_Time	0.012	0.015	0.017	0.016	0.005	0.008	0.047 **	0.044 **
	(0.014)	(0.014)	(0.013)	(0.013)	(0.008)	(0.008)	(0.017)	(0.017)
	**Panel D: Unhealthy Weight-loss Methods**							
LoseWeight_Fasting	0.011	0.009	0.028 **	0.028 **	0.110 **	0.113 **	0.082 **	0.085 **
	(0.011)	(0.011)	(0.007)	(0.007)	(0.007)	(0.007)	(0.01)	(0.009)
LoseWeight_Med	0.055 **	0.053 **	0.024 **	0.024 **	0.090 **	0.092 **	0.062 **	0.060 **
	(0.010)	(0.010)	(0.006)	(0.006)	(0.007)	(0.007)	(0.008)	−00.008

Bootstrapped standard errors are in parentheses. Asterisks, ** and *, indicate the 1%, and 5% significance level, respectively.

Third, the original treatment group consists of adolescents who perceive themselves as either slightly or significantly overweight. One concern is that adolescents in the treatment group who deem themselves to be slightly overweight can be significantly similar to the comparison group and, therefore underestimate the treatment effects. We define an alternative treatment group that only consists of adolescents who think themselves as significantly overweight. As shown in Table 9, the effects on weight-loss intentions as well as on eating and exercise habits are similar to those for the original treatment group. The only exception is that self-perception of being significantly overweight does not cause normal-weight males to have a stronger intention to lose weight through exercise. Compared with the main results, we also observe a much larger treatment effect of overweight perception on extreme weight-loss methods for normal-weight adolescents. This finding indicates that a stronger bias in self-perception of being overweight could be more harmful through the adoption of extreme weight-loss methods.

Fourth, researchers have found that adolescents underreport their weights and overreport their heights, which leads to self-reported measurement errors in BMI [51]. Following Fan (2010), we correct for self-reported BMI by using the National Health and Nutrition Examination Survey 1999–2008 that have both self-reported and doctor-measured weights and heights [52]. The results based on the corrected BMI rather than the self-reported BMI are highly similar to the main results. Details about the estimation of correction equations are provided in Appendix C.

Finally, we also estimate the effects using a parametric two-stage treatment model (see Maddala (1983) for details [53]). As shown in Table 10, the estimated marginal effects of self-perception of being overweight are qualitatively consistent with the main results.

**Table 9:** Effects of Self-perception of Being *Significantly* Overweight on Weight-loss Intentions and Behaviors using Propensity Score Matching.

	Self-Reported Overweight Sample				Self-Reported Normal-Weight Sample			
	Female		Male		Female		Male	
	NNM	LLR	NNM	LLR	NNM	LLR	NNM	LLR
	**Panel A: Weight-loss Intentions**							
LoseWeight	0.129 **	0.154 **	0.310 **	0.308 **	0.335 **	0.347 **	0.362 **	0.404 **
	(0.025)	(0.025)	(0.035)	(0.028)	(0.038)	(0.022)	(0.088)	(0.077)
LoseWight_Diet	0.136 **	0.161 **	0.161 **	0.182 **	0.314 **	0.333 **	0.388 **	0.367 **
	(0.038)	(0.035)	(0.037)	(0.036)	(0.038)	(0.027)	(0.085)	(0.069)
LoseWeight_Exer	0.043	0.065 *	0.081 *	0.065 *	0.137 **	0.158 **	0.019	0.091
	(0.032)	(0.028)	(0.032)	(0.028)	(0.035)	(0.027)	(0.097)	(0.073)
	**Panel B: Eating Habits**							
Fruit_2	−0.010	−0.007	−0.052	−0.054	0.021	0.036	0.063	0.050
	(0.033)	(0.028)	(0.029)	(0.028)	(0.040)	(0.034)	(0.079)	(0.060)
Vegetable_3	−0.023	−0.017	−0.017	−0.031 *	0.036	0.032	0.071	0.098
	(0.019)	(0.018)	(0.017)	(0.015)	(0.023)	(0.019)	(0.065)	(0.058)
Soda	−0.083	−0.087	−0.038	−0.034	−0.074	−0.068	−0.008	0.024
	(0.068)	(0.088)	(0.057)	(0.057)	(0.065)	(0.055)	(0.130)	(0.092)
	**Panel C: Physical and Sedentary Activity**							
Active_5Days	−0.009	0.005	−0.067	−0.069	0.010	0.015	0.006	−0.036
	(0.048)	(0.039)	(0.046)	(0.042)	(0.058)	(0.046)	(0.120)	(0.095)
Moderate_Exer	0.012	0.023	−0.113 **	−0.115 **	0.065	0.043	−0.216 *	−0.234 **
	(0.036)	(0.038)	(0.035)	(0.032)	(0.047)	(0.032)	(0.097)	(0.075)
Vigorous_Exer	−0.031	−0.024	−0.043	−0.026	0.065	0.052	−0.020	−0.025
	(0.033)	(0.031)	(0.034)	(0.029)	(0.041)	(0.031)	(0.086)	(0.061)
PE_Class	−0.017	−0.015	−0.13 **	−0.106 **	−0.013	−0.001	−0.127	−0.108
	(0.037)	(0.034)	(0.032)	(0.03)	(0.044)	(0.034)	(0.090)	(0.067)
Sport_Team	−0.038	−0.031	−0.164 **	−0.155 **	−0.038	−0.050	−0.224 *	−0.228 **
	(0.036)	(0.031)	(0.032)	(0.029)	(0.052)	(0.037)	(0.099)	(0.076)
TV_Time	0.049	0.031	0.035	0.046	0.052	0.022	0.165	0.185 *
	(0.038)	(0.035)	(0.039)	(0.034)	(0.046)	(0.033)	(0.096)	(0.083)
Video_Time	−0.013	0.005	0.054	0.040	0.014	0.019	0.111	0.138
	(0.042)	(0.043)	(0.044)	(0.038)	(0.045)	(0.038)	(0.115)	(0.087)
	**Panel D: Unhealthy Weight-loss Methods**							
LoseWeight_Fasting	0.002	0.006	0.025	0.028	0.294 **	0.300 **	0.296 **	0.294 **
	(0.038)	(0.034)	(0.024)	(0.024)	(0.044)	(0.033)	(0.068)	(0.064)
LoseWeight_Med	0.055	0.057 *	0.062 **	0.049 *	0.278 **	0.295 **	0.353 **	0.357 **
	(0.038)	(0.034)	(0.024)	(0.024)	(0.044)	(0.033)	(0.068)	(0.064)

Bootstrapped standard errors are in parentheses. Asterisks, ** and *, indicate the 1%, and 5% significance level, respectively.

**Table 10:** Marginal Effects of the Perception of Being Overweight on Weight-loss Intentions and Behaviors based on the Two-stage Treatment Model.

	Female		Male	
	RNOS	ROS	RNOS	ROS
	**Panel A: Weight-loss Intentions**			
LoseWeight	0.346 ***	0.239 ***	0.574 ***	0.406 ***
	(0.027)	(0.011)	(0.027)	(0.003)
LoseWight_Diet	0.305 ***	0.162 ***	0.424 ***	0.237 ***
	(0.034)	(0.019)	(0.041)	(0.003)
LoseWeight_Exer	0.109 ***	0.093 ***	0.240 ***	0.138 ***
	(0.019)	(0.015)	(0.004)	(0.003)
	**Panel B: Eating Habits**			
Fruit_2	−0.015 ***	−0.010 ***	−0.017 ***	−0.043 ***
	(0.001)	(0.002)	(0.003)	(0.004)
Vegetable_3	0.000	−0.013 **	−0.003	−0.023 ***
	(0.002)	(0.006)	(0.009)	(0.002)
Soda	−0.022	−0.030 ***	0.015	−0.014 ***
	(0.014)	(0.003)	(0.046)	(0.005)
	**Panel C: Physical and Sedentary Activity**			
Active_5Days	−0.036 ***	−0.026 **	−0.128 ***	−0.122 ***
	(0.002)	(0.012)	(0.028)	(0.012)
Moderate_Exer	−0.007	0.003	−0.056 ***	−0.055 ***
	(0.004)	(0.008)	(0.003)	(0.003)
Vigorous_Exer	−0.013 **	0.002	−0.130 ***	−0.054 ***
	(0.005)	(0.019)	(0.047)	(0.008)
PE_Class	−0.028 ***	−0.007	−0.086 ***	−0.066 ***
	0.000	(0.007)	(0.019)	(0.007)
Sport_Team	−0.019 **	−0.004	−0.147 ***	−0.120 ***
	(0.010)	(0.003)	(0.038)	(0.013)
TV_Time	−0.016 ***	−0.001	0.042 ***	0.045 ***
	(0.002)	(0.003)	0.000	(0.006)
Video_Time	0.004	0.000	0.139 ***	0.012 ***
	(0.017)	(0.014)	(0.050)	(0.004)
	**Panel D: Unhealthy Wight-loss Methods**			
LoseWeight_Fasting	0.548 ***	0.027	0.390 ***	0.026 ***
	(0.041)	(0.031)	(0.031)	(0.001)
LoseWeight_Med	0.405 ***	0.054 ***	0.082 ***	0.018 ***
	(0.032)	(0.018)	(0.020)	(0.002)

Bootstrapped standard errors are in parentheses. Asterisks, ***, ** and *, indicate the 1%, 5% and 10% significance level, respectively.

## 5. Conclusions

This study analyzes the effects of self-perception of being overweight on weight-loss intentions and behaviors among U.S. adolescents. The main findings are based on a semi-parametric technique, propensity score matching, with a series of robustness checks. We find significant discrepancies between weight-loss intentions and behaviors. Irrespective of self-reported weight status, adolescents who perceive themselves as overweight have a stronger intention to lose weight (19–55 percentage points higher for the general weight-loss intention as well as 15–31 and 8–15 percentage points higher for losing weight through diet and exercise, respectively), yet, the increased intention does not transfer into improvements in eating and exercise habits by consuming at least the recommended levels of fruits and vegetables, less soft drinks, or become physically more active. Instead, adolescents with overweight perception engage in extreme weight-loss methods to gain immediate gratification, especially for normal-weight adolescents (9–11 percentage points for fasting, 6–10 percentage points for unguided diet medication).

To stop or even reverse the rising trend of childhood obesity, we have to find effective strategies to ease psychological and behavioral barriers and modify adolescents’ diet and physical habits. Many empirical studies have documented that people have time-inconsistent preferences and self-control problems [54,55,56], which lead to a gap between long-run intentions and short-run actions. People who have time-inconsistent preferences are likely to procrastinate eating healthy and exercising [47,49]. Whitlock *et al.*, find that the short-lived effect of intervention programs can be partly explained by the incapability of transforming intentions into actual behavior in an uncontrolled environment once the programs are completed [57]. Having incorrect self-perception of weight status could potentially exacerbate their self-control problem and prevent them from adopting healthy life style. For overweight adolescents who fail to recognize their weight problems, motivating them to eat healthy or exercise might become more challenging. For normal-weight adolescents who are obsessed with slender body, they prefer extreme weight-loss methods with immediate effects rather than improving their eating and exercise habits. There is a great need to provide interventions to help adolescents overcome their impatience and enhance self-control. For example, nudging in a variety of formats has been used in both school and shopping environments to encourage individuals to either purchase and/or consumer more fruits and vegetables [58,59]. Another self-perception relevant barrier found to constrain physical activity participation among overweight adolescents is the feeling of “too fat to exercise” or “behavioral incapability” [60]. Under such circumstance, interventions might focus on making adolescents gain confidence and think they are indeed capable of engaging in physical activities at an appropriate level even if they are overweight or obese. This study provides evidence that behavior and psychological management skills are required for the success of behavior intervention programs and that policies targeting childhood obesity through changing adolescents’ behavior requires incorporation of partial or full irrationality rather than rationality.

We envision two directions for future research but different data sets are needed. First, the YRBSS does not have information about adolescents’ family and friends. Adolescents’ self-perception of body weight and their eating and exercise habits are likely influenced by family food environment, parental attitude and preference relating to eating and exercise habits, and their friends and peers. Future research incorporating family and neighborhood environment is warranted. Second, we are not able to examine the dynamic decisions over time since the YRBSS is repeated cross-section data. Understanding and modeling the inter-period decision making process of adolescents might be a fruitful research area if data are available in the future.

## Figures and Tables

**Figure A1:**
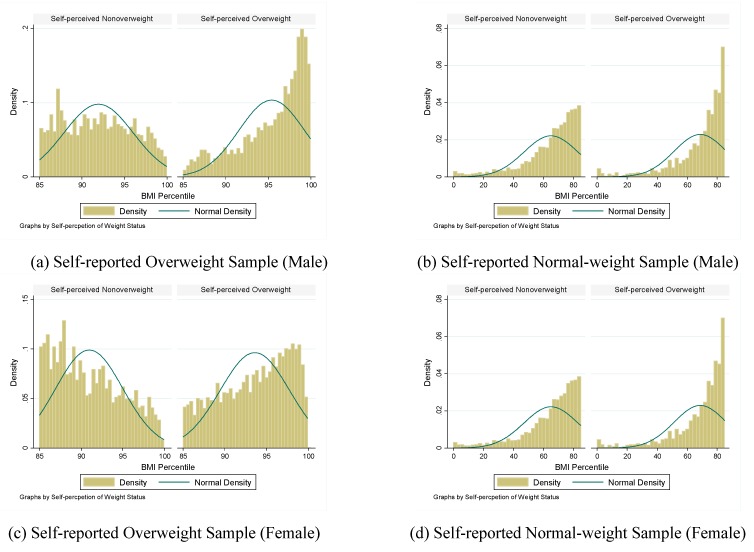
Overlap of the BMI Percentile between the Treatment and Comparison Groups.

**Figure A2:**
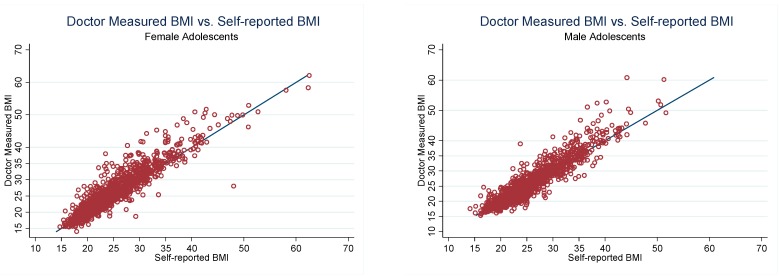
Self-reported BMI versus Doctor Measured BMI.

## Appendix

### Appendix A. Survey Questions and Variables Created Based on These Survey Questions

**Table A1:** Survey Questions and Variables Created.

Survey Question	Choice Options	Variable Created
During the past 7 days, how many times did you eat fruits?	(a) I did not eat fruits during the past 7 days. (b) 1 to 3 times during the past 7 days (c) 4 to 6 times during the past 7 days (d) 1 time per day (e) 2 times per day (f) 3 times per day (g) 4+ times per day	*Fruit*_2 = 1 if the respondent eat fruits at least 2 times a per day in the past 7 days.
During the past 7 days, how many times did you eat vegetables?	(a) I did not eat fruits during the past 7 days. (b) 1 to 3 times during the past 7 days (c) 4 to 6 times during the past 7 days (d) 1 time per day (e) 2 times per day (f) 3 times per day (g) 4+ times per day	*Veg*_3 = 1 if the respondent eat vegetables at least 3 times per day in the past 7 days.
During the past 7 days, how many times did you drink a can, bottle, or glass or soda or pop, such as Coke, Pepsi, or Sprite? (Do not include diet soda or diet pop.)	(h) I did not drink soda or pop during the past 7 days. (i) 1 to 3 times during the past 7 days (j) 4 to 6 times during the past 7 days (k) 1 time per day (l) 2 times per day (m) 3 times per day (n) 4+ times per day	*Soda* = 1 if the respondent drink at least one soda or pop per day in the past 7 days.
During the past 7 days, on how many days were you physically active for a total of at least 60 min per day? (Add up all the time you spent in any kind of physical activity that increased your heart rate and made your breather hard some of the time.)	Choice options ranging from zero to 7 days.	*Active_5Days* = 1 if the respondent reported 5 days he/she were physically active.
On how many days of the past 7 days did you exercise or participate in physical activity for at least 30 min that do not made you sweat and breathe hard?	Choice option ranging from zero to 7 days.	*Moderate_Exer* = 1 if the respondent reported at least three days for such vigorous activity.
On how many days of the past 7 days did you exercise or participate in physical activity for at least 20 minutes that made your sweat and breathe hard, such as basketball, soccer, running, swimming laps, fast bicycling, fast dancing, or similar aerobic activities?	Choice option ranging from zero to 7 days.	*Vigorous_Exer* = 1 if the respondent reported at least three days for such vigorous activity.
How many days per week do you usually go to physical education (PE)?	Choice options ranging from zero to 5 days.	*PE_class* = 1 if the respondent had at least one day attending PE.
During the past 12 months, on how many sports teams did you play? (Include any teams run by your school or community groups.)	Choice options : zero team, 1 team, 2 teams, and 3+ teams	*Sport_Team* = 1 if the respondent played at least one team.
On an average school day, how many hours do you watch TV?	(a) I do not watch TV on average school day. (b) 1 hour per day. (c) 2 hours per day (d) 3 hours per day (e) 4 hours per day (f) 5+ hours per day	*TV_time* = 1 if the respondent watched TV for at least three hours on an average school day.
On an average school day, how many hours do you play video or computer games or use a computer for something that is not school work.	(a) I do not play video or computer games or use a computer for something that is not school work. (b) 1 hour per day. (c) 2 hours per day (d) 3 hours per day (e) 4 hours per day (f) 5+ hours per day	*Video_time* = 1 if the respondent paly video or computer games or use a computer for something that is not school work for at least three hours per day.
Which of the following are you trying to do about your weight?	(a) Lose weight (b) Gain weight (c) Stay the same weight (d) I am not trying to do anything about my weight.	*LoseWeight* = 1 if the respondent chooses option b).
During the past 30 days, did you diet to lose weight or to keep from gaining weight?	(e) Yes (f) No	*LoseWeight_Diet* = 1 if the respondent chose option a)
During the past 30 days, did you exercise to lose weight or to keep from gaining weight?	(g) Yes (h) No	*LoseWeight_Exer* = 1 if the respondent chose option a)
During the past 30 days, did you go without eating for 24 hours or more (also called fasting) to lose weight or to keep from gaining weight?	(i) Yes (j) No	*LoseWeight_Fasting* = 1 if the respondent chose option a)
During the past 30 days, did you take any diet pills, powder, or liquids without a doctor’s advice to lose weight or to keep from gaining weight?	(k) Yes (l) No	*LoseWeight_Med* = 1 if the respondent chose option a)

### Appendix C. Correction of Self-Reported Height and Weight

Measurement error resulting from the self-reported height and weight in YRBSS data could potentially bias our estimates, especially for underweight and overweight adolescents. The National Health and Nutrition Examination Survey (NHANES) is so far the best source to study the measurement error of self-reported height and weight as it have both self-reported and doctor measured height and weight.

Figure A1 plots the self-reported BMI and the doctor-measured BMI using the NHEANS adolescent sample aged 16 to 18 since self-reported height and weight are not available for adolescents aged 14 or 15 in NHANEs, where the solid line is 45-degree. It clearly shows discrepancies between the two BMI measures.

Using the NHEANS adolescent sample, we conduct an ordinary linear regression in which the dependent variable is the doctor measured BMI value and the independent variables consists of the reported BMI value and other covariates. We then predict the BMI for the YRBSS respondents using the relevant variables in the YRBSS data.
